# Stable expression and functional characterisation of the diamondback moth ryanodine receptor G4946E variant conferring resistance to diamide insecticides

**DOI:** 10.1038/srep14680

**Published:** 2015-10-01

**Authors:** Bartlomiej J. Troczka, Alan J. Williams, Martin S. Williamson, Linda M. Field, Peter Lüemmen, T.G. Emyr Davies

**Affiliations:** 1 Biological Chemistry and Crop Protection Department, Rothamsted Research, Harpenden, Hertfordshire, AL5 2JQ, UK; 2 Institute of Molecular & Experimental Medicine, Cardiff University School of Medicine, Wales Heart Research Institute, Heath Park, Cardiff CF14 4XN, UK; 3 Bayer CropScience AG, 65926 Frankfurt, Germany

## Abstract

Diamides, such as flubendiamide and chlorantraniliprole, belong to a new chemical class of insecticides that act as conformation-sensitive activators of insect ryanodine receptors (RyRs). Both compounds are registered for use against lepidopteran species such as the diamondback moth, *Plutella xylostella*, a notorious global pest of cruciferous crops. Recently acquired resistance to diamide insecticides in this species is thought to be due to a target-site mutation conferring an amino acid substitution (G4946E), located within the trans-membrane domain of the RyR, though the exact role of this mutation has not yet been fully determined. To address this we have cloned a full-length cDNA encoding the *P. xylostella* RyR and established clonal Sf9 cell lines stably expressing either the wildtype RyR or the G4946E variant, in order to test the sensitivity to flubendiamide and chlorantraniliprole on the recombinant receptor. We report that the efficacy of both diamides was dramatically reduced in clonal Sf9 cells stably expressing the G4946E modified RyR, providing clear functional evidence that the G4946E RyR mutation impairs diamide insecticide binding.

Ryanodine receptors (RyRs) are tetrameric calcium-activated calcium release channels located on the endoplasmic reticulum. They are responsible for the control of calcium release from internal stores and are primarily known for their role in excitation-contraction coupling in muscle cells. They are the largest ion channels known, comprising circa 5000 amino acids per subunit^1^. There are 3 RyR isoforms characterized in mammals (RyR1–3) but only one in invertebrates, with approximately 45–47% homology to the mammalian counterpart(s)^2^. RyRs were considered a viable insecticide target for many decades; however several attempts at creating a commercially successful compound based on the chemical structure of ryanodine, a universal RyR modulator and well known pharmacological probe, ultimately failed due to unacceptable levels of toxicity in non-target organisms^3^. More recently, a new class of synthetic RyR activators, the diamides (IRAC mode of action classification Group 28 insecticides^4^), was successfully commercialized. They were first introduced to the market with the release of the phthalic acid diamide, flubendiamide (Bayer CropScience) in 2006^5^ followed by the anthranilic acid diamide, chlorantraniliprole (DuPont Crop Protection, USA) in 2007^6^. The new chemistries exhibited high potency against lepidopteran species such as *Plutella xylostella* and an excellent toxicological profile^7^,^8^ as well as no established cross-resistance with any other commercially available compounds^9^. Since their launch in 2006, the sale of diamides has increased substantially, currently accounting for 8% of the global insecticide market^10^, and over that period these insecticides have become one of the primary chemical control agents against lepidopteran pests due to their favourable biological, ecological and toxicological attributes^6^,^11^.

The diamide insecticides flubendiamide and chlorantraniliprole both have a similar mode of action, and act to prolong RyR channel opening, resulting in uncoordinated muscle contractions in intoxicated pest insects^11^,^12^. Due to their novelty, relatively little is known about how diamides interact with insect RyRs and how their high insecticidal specificity is achieved. Studies with chimeric proteins (insect-mammal and insect-nematode chimeras) pinpointed the probable location of diamide binding within the transmembrane region of the receptor^13^,^14^. However there is growing evidence for different diamide-binding profiles across various insect orders^15^,^16^,^17^, suggesting there may be structural variability within the binding region.

The first diamide control failures were reported for the diamondback moth, *P. xylostella,* one of the most destructive pests of cruciferous crops, accounting for $4-5 billion pounds worth of annual worldwide crop losses^18^, and being the most extensively distributed of all Lepidoptera globally^19^. Initial reports of resistance came from the Bang Bua Thong district of Thailand just 18 months after flubendiamide was launched. The following year (2010) *P. xylostella* resistance to diamides was reported in Cebu, Philippines and in 2011 in Yin Ling and Chang Hwa, Taiwan and in the Guangzhou and Guandong provinces of Southern China^20^. Reports of resistance were followed by the discovery of a G4946E amino acid substitution (Fig. 1) within the transmembrane spanning region of RyR of highly resistant populations of *P. xylostella* from Thailand (Bang Bua Thong) and the Philippines (Sudlon, Cebu)^21^. The same mutation was later independently found in diamide resistant populations from China^22^. Binding studies to native membranes isolated from resistant populations collected from Southern China with fluorescently labelled diamide probes indicated a decreased affinity for diamides in resistant individuals^23^. However, the study largely failed to provide convincing functional evidence that the G4946E mutation confers a significant level of target-site resistance.

In this present study we report the cloning, characterization and successful expression of the full-length *P. xylostella* RyR. The cell lines expressing the receptor were used to conduct a more in-depth analysis of the effects of the G4946E substitution on receptor sensitivity to diamide insecticides and on general channel function.

## Results

### Cloning and characterisation of a functional RyR from *P. xylostella*

The cloned *P. xylostella* RyR cDNA in this study encodes a 5118 amino acid protein (NCBI accession AFW97408), with a high degree of similarity to other successfully expressed insect RyRs including those from *Drosophila melanogaster* (78.5%) and *Bombyx mori* (91.5%)^13^,^24^. It shares all of the features described previously for the RyR isolated from *P. xylostella* in other studies^25^,^26^,^27^, including a GXRXGGGXGD selectivity filter motif in the transmembrane domain and MIR (212–393), SPRY (664–803, 1091–1214, 1551–1693) and RIH domains (2232–2454) in the cytosolic region. The lepidopteran specific amino acids at positions N(4953), N(4955), N(4966), L(4981), L(5012), N(5044), T(5095) are also present^28^. Overall a large number (over 200) of silent SNPs were found within our wild type version of the RyR (ROTH WT), with a clustered distribution (Fig. 2b). Four alternative splice sites were identified, three of which correspond to previously described splice sites IS2, IS3 and IS10 in *Wang et al.*^26^ who also reported an additional seven splice variants. Multiple alignments of the *P. xylostella* RyR sequences with other insect RyRs suggest that the alternative splice forms identified in the moth are most likely not representative of the prevalent form of the receptor. This hypothesis is supported by the report of alternative splicing frequency published by *Wang et al.*^26^. Similar polymorphism in a lepidopteran RyR gene was also described in the tobacco budworm *Heliothis virescens*^29^.

### Functional analysis of transiently expressed RyR channels

*Spodoptera frugiperda* Sf9 cells transformed with the *P. xylostella* ryanodine receptors were loaded with FURA2, a fluorescent Ca^2+^ indicator, and were exposed to caffeine (30 mM), followed by high concentrations of flubendiamide or its somewhat more water soluble analogue flubendiamide sulfoxide (Fig. 3). Sf9 cells expressing the ROTH WT or the G4946E RyR were able to respond to repeated caffeine applications, as demonstrated by a brief elevation of intracellular Ca^2+^ concentration measured at 72 h post transfection. Flubendiamide sulfoxide also showed reversible activation of the calcium release in cells expressing ROTH WT RyR. Conversely, exposure of the ROTH WT RyR cells to flubendiamide resulted in permanently increased Ca^2+^ levels and abolished any further caffeine evoked activation for the duration of the experiment, suggesting a fixing of the receptor in a permanently open state. Sf9 cells expressing the G4946E RyR variant did not show any calcium release when exposed to flubendiamide (Fig. 3). The more water soluble analogue, flubendiamide sulfoxide also failed to induce any release of calcium (data not shown). Additionally, unlike ROTH WT RyR cells, the G4946E variant remained sensitive to caffeine after exposure to flubendiamide. In the control un-transfected cells and cells exposed only to Cellfectin, caffeine and diamides failed to evoke any such responses.

Tritium labelled ryanodine binding experiments on isolated mixed-membrane preparations from Sf9 cells transiently expressing ROTH WT or G4946E RyRs gave B_max_ and K_d_ values of 2447 ± 425 dpm (244.9 ± 42.5 fmol/mg) and 12.89 ± 4.5 nM for WT and 2671 ± 401 dpm (267 ± 40 fmol/mg) and 14.69 ± 4.18 nM for the G4946E variant (Fig. 4). The K_d_ values for both constructs were higher than seen for native preparations of rabbit skeletal muscle RyR (2.04 ± 0.12 nM) and *H. virescens* RyR (3.82 ± 0.39 nM), which also have much higher B_max_ values; 4.57 ± 0.32 pmol/mg (rabbit) and 2.41 ± 0.17 pmol/mg (*H. virescens*)^30^. However in a different study of *H. virescens* native membrane preparations, the respective K_d_ value was 13.9 ± 3.8 nM, making it comparable to the Sf9 expressed *P. xylostella* RyRs, while the B_max_ values were between 1–1.5 pmol/mg^31^. Apparent differences in K_d_ and B_max_ with those reported in other studies could likely be explained by the different experimental conditions. It is clear that the presence of the G4946E mutation does not appear to have any significant effects on the *P. xylostella* RyRs ability to bind ryanodine, since both K_d_ and B_max_ values are similar.

### Functional analysis of stably expressed RyR channels

Single Sf9 cell clones stably expressing either the wild-type (Sf9-wtRyR) or the G4946E (Sf9-mRyR) RyR channel were isolated after a few cycles of clonal selection. Intracellular calcium signals induced by 30 mM caffeine in Sf9-wtRyR increased rapidly to the maximum in less than one minute after application and then decreased to baseline levels (Fig. 5). The transient nature of the calcium signals induced by millimolar caffeine concentrations was comparable to those measured previously in isolated *H. virescens* neurons^31^. This may be explained by compensatory calcium re-uptake mechanisms of the sarco-/endoplasmic reticulum, in particular the Ca^2+^-ATPase (SERCA)^32^,^33^. The longer time scale of the caffeine-induced calcium transients in the Sf9-wtRyR cells, as compared to the isolated *H. virescens* neurons may indicate that excitable cells such as neurons and muscle cells can fine-tune intracellular calcium concentrations more efficiently than can the Sf9 cells lacking specific RyR accessory proteins.

In contrast to caffeine, the diamide insecticides chlorantraniliprole and flubendiamide-sulfoxide evoked increased intracellular calcium concentrations that persisted during the time period of the measurement. This may be explained by the mechanistic hypothesis that diamides preferentially bind to the calcium-conducting conformational state of the RyR and stabilize the open channel due to the low nanomolar equilibrium dissociation constant of the diamide-RyR complex^34^.

Measurements of the integrated calcium signals as a function of the diamide concentration, gave characteristic dose-response curves, from which apparent EC_50_ concentrations for flubendiamide-sulfoxide and chlorantraniliprole were calculated (Fig. 6). These values represent the effector concentration causing half-maximal integrated calcium signals in the cells. The EC_50_ of flubendiamide sulfoxide was 245 nM± 46 nM (standard error) and the EC_50_ of chlorantraniliprole was 17 nM± 2 nM (standard error). It should be noted that the EC_50_ values are not only determined by the compound’s affinities to the RyR, but also by their availability at the intracellular target site as influenced by their physico-chemical properties.

The efficacy of both diamides was reduced dramatically in the Sf9-mRyR cells expressing the G4946E RyR. It was possible to measure a full dose-response curve of chlorantraniliprole on the G4946E RyR, from which an EC_50_ value of 3715 nM± 776 nM (standard error) was calculated. The limited solubility of the flubendiamide sulfoxide at concentrations exceeding 20 μM prevented the measurement of complete dose-response curves and, consequently, an accurate EC_50_ value could not be determined beyond an approximation of >20,000 nM.

The results show that chlorantraniliprole had roughly two orders of magnitude less effect on the *P. xylostella* RyR channel with the G4946E substitution, indicating a drastic reduction of the receptor´s affinity to the diamide and making it very likely that the G4946E substitution is responsible for the observed resistance to diamides in *P. xylostella.*

## Discussion

Diamide insecticides such as chlorantraniliprole and flubendiamide are a new class of insecticide recently introduced to the market to control a broad range of herbivorous pest insects, particularly of the order Lepidoptera. An over reliance on the use of diamide insecticides against diamondback moth has led to a rapid resistance development, particularly in Asia where the field efficacy of these compounds has now been seriously compromised. Larvae collected from the Philippines and Thailand in 2012 were found to be over 200-fold resistant to both chlorantraniliprole and flubendiamide compared to susceptible strains^21^. Non-synonymous mutations in each of the resistant strains that in both cases lead to a glycine to glutamic acid substitution (G4946E) in the protein were identified. The independent evolution of the same amino acid substitution, within a highly conserved region of the RyR channel, the C-terminal membrane-spanning domain, in two geographically separated resistant strains of *P. xylostella* strongly suggested a causal association with diamide resistance. The same mutation was subsequently independently identified in field populations of *P. xylostella* from China, 303–658 fold resistant to chlorantraniliprole^23^. A recent genotyping study^35^ has confirmed the global presence of the G4946E mutation in ten different countries where diamide insecticides have largely failed to control diamondback moth populations, a significant correlation further highlighting the likely role of this target-site mutation in conferring resistance to diamides.

Radioligand binding studies with a tritiated flubendiamide derivative [^3^H] PAD1 using *P. xylostella* thoracic microsomal membrane preparations from susceptible and resistant (Philippine) strains^35^ has provided further compelling evidence for the involvement of the RyR G4946E mutation on both diamide specific binding and its concentration dependent allosteric modulation of [^3^H] ryanodine binding^17^,^34^. In contrast to the susceptible strain, with reported K_d_ and B_max_ values of 2.7 ± 0.23 nM and 8.3 ± 0.19 pmol mg^−1^, for the Philippine strain saturation binding with the tritiated flubendiamide analogue was not reached and meaningful equilibrium kinetics could not be calculated, suggesting that the G4946E mutation confers target-site resistance to diamide insecticides. EC_50_ values for ryanodine binding stimulation in the Philippine strain were at least 100-fold higher, with resistance ratios of >450 and 159 fold for flubendiamide and chlorantraniliprole, respectively. Reciprocal crosses of the Philippine strain, homozygous for G4946E, with a susceptible laboratory strain yielded F1 progeny with a diamide susceptible phenotype, suggesting an autosomal, recessive mode of inheritance. Subsequent back-crosses with the parental lines indicated a near monogenic inheritance for diamide resistance in the Philippine strain. The data reported in this current study, whereby we directly investigated binding of diamide insecticides to recombinant RyR proteins, now unambiguously provides clear functional evidence that the G4946E RyR mutation greatly impairs diamide insecticide binding and is responsible for the resistant phenotype exhibited by the resistant *P. xylostella* strains.

**Figure 1:**
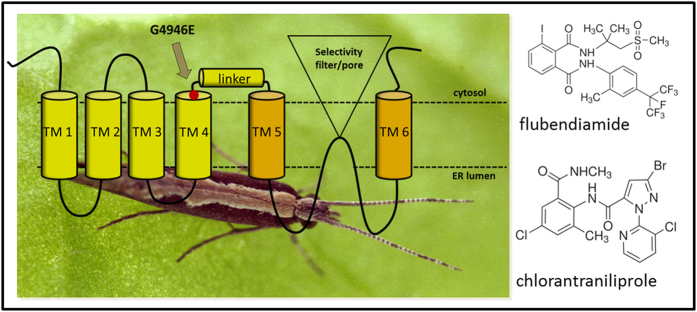
Transmembrane protein topology of the C-terminal domain of the *P. xylostella* RyR based on the crystal structure of rabbit RyR1^36^. The region containing the G4946E substitution (position G4900 in published sequence^21^, accession number JX467684) links predicted membrane-spanning domains TM4 and TM5. The chemical structures of flubendiamide and chlorantraniliprole are also shown. Image © Rothamsted Research Ltd.

Mapping of the G4946E substitution onto the recently published high resolution crystal structure of the (closed state) rabbit RyR1 channel^36^,^37^ suggests that this residue in the WT *P. xylostella* channel potentially acts as a glycine hinge at the interface between the transmembrane spanning S4 helix and the S4-S5 linker domain (Fig. 1), hence residue changes at this position are likely to have a major impact on movement of the S5 and S6 helices that control opening and closing of the channel pore, and have a direct knock on effect on binding of diamide insecticides to the receptor. Three additional point substitutions (E1338D, Q4594L and I4790M) in the *P. xylostella* RyR were recently reported in a field population collected from Yunnan province, China^38^ (corresponding to residues E1333, Q4548, I4744 in the channel expressed in this current paper), exhibiting up to 2128-fold resistance to diamide insecticides. It has been shown that the I4790M change (located midway along the RyR S2 helix) may lie in close proximity to the G4946 residue in the 3D structure of the RyR^35^ and consequently these two residues may define the diamide binding site on the receptor. The isoleucine residue at position 4790 is specific to Lepidoptera, being a methionine in all other insects, spiders and mites, a leucine in nematodes and a cysteine in mammalian RyRs, so this residue may be responsible for the differential sensitivities of the *P. xylostella* and other insect RyR channels to flubendiamide and chlorantraniliprole chemistries^16^,^17^.

Chlorantraniliprole and flubendiamide have also been extensively used to manage the tomato leaf miner *Tuta absoluta* (Lepidoptera: Gelechiidae), an invasive pest of tomato crops that is rapidly expanding around the globe. High resistance levels of up to 2,414- and 1,742-fold for chlorantraniliprole and flubendiamide, respectively have been detected in a *T. absoluta* population originating from Sicily (Italy), suggestive of a similar target-site insensitivity in this species^39^. Cross resistance between flubendiamide and chlorantraniliprole has also recently been reported for the smaller tea tortrix, *Adoxophyes honmai*^40^, with resistance ratios of 105-fold and 77.2-fold respectively. Even though the level of relative activity by resistant individuals may vary among the two actives, ultimately the result is inadequate field control. Currently, resistant populations in most countries are still fairly localised and many crop production areas will still maintain susceptible populations. Significant resistance management efforts are therefore needed to protect diamide chemistry as a useful pest management tool, especially with the introduction over the next few years of additional manufacturers competitively selling multiple brands containing different diamide actives. It has been recognised that there will need to be a concerted and coordinated programme of insect monitoring for early detection of diamide tolerance within populations, coupled with the use of appropriate insecticide rotations with different modes of action chemistries, to alleviate this emerging problem^41^. The cell lines stably expressing RyR variants generated in this study also offer opportunities for a high throughput counter-screening of chemical libraries to seek for compounds overcoming the resistance.

## Methods

### Chemicals

All stand-alone chemicals used for the preparation of bacterial media were purchased from Sigma. The DMSO used for dilution of all active compounds was of analytical grade (purity ≥ 99%) and supplied by Sigma. [^3^H] ryanodine (spec. activity 2 TBq mmol^−1^) was supplied by PerkinElmer. Flubendiamide, flubendiamide sulfoxide and chlorantraniliprole were of technical grade (purity > 98%) provided in-house (Bayer CropScience) and purchased as analytical standard from Fluka Chemicals (Buchs, Switzerland), respectively. Analytical grade caffeine was from ReagentPlus® (Sigma).

### *P. xylostella* strains

The diamide susceptible *P. xylostella* laboratory strain (ROTH), originally collected in the UK, has been reared on Chinese cabbage plants (*Brassica rapae Spp*) for over 40 years under controlled environmental conditions. The maintenance and sequencing of the resistant (G4946E) strain is described elsewhere^21^.

### RNA extractions and cDNA synthesis

Total RNA was extracted from 35 mg of flash frozen *P. xylostella* larvae using an E.Z.N.A® Mollusc RNA kit (Omega Bio-Tek, GA, USA), following the manufacturer’s guidelines. 4 μg of total RNA, 100 pmol of an Oligo (_d_T)25 primer and 2 μl dNTPs (10 mM) were used for cDNA synthesis with 200 U of RevertAid premium (Thermo) reverse transcriptase in 20 μl reactions. The cDNA synthesis reactions were incubated at 55 °C for 1 h.

### Cloning of *P. xylostella* RyR

Initially 3 small (approx. 500–1000 bp) fragments of the RyR were PCR amplified, using degenerate primer pairs based on the silkworm *B. mori* RyR sequence (NCBI accession number XM_004924859.1). Gene specific primers were then designed (based on the sequence information obtained from these fragments), and two large fragments, spanning the approximately 7 Kb gaps between the 3 initial fragments, were amplified, cloned and sequenced. The extreme 5′ and 3′ ends of the receptor were obtained through RACE.

**Figure 2:**
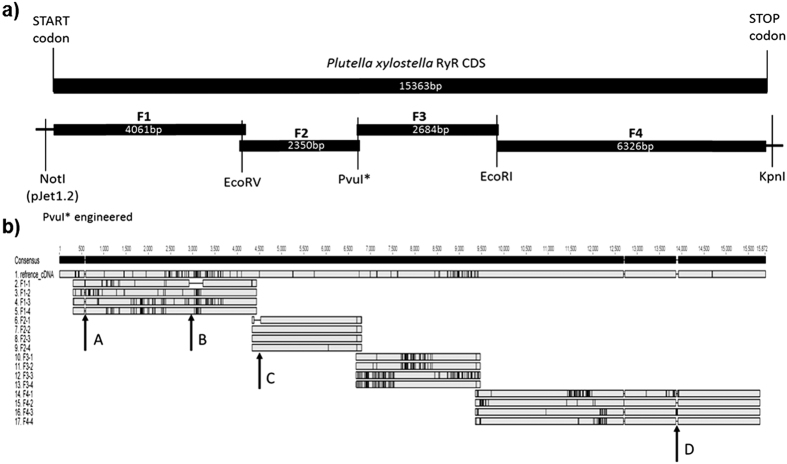
(**a**) Strategy employed for the assembly of the full-length *P. xylostella* RyR. The *Not*I restriction site needed for the final assembly came from the pJet1.2 vector used for cloning of individual fragments. (**b**) Alignment of sub-cloned *P. xylostella* WT RyR fragments against the initial cDNA reference showing the position (black vertical lines) corresponding to individual SNP variants. The approximate location of four identified splice sites A–D are indicated by black arrows. Splice sites A, C and D were described in Wang *et al.*^26^, whereas splice site B was not reported.

**Figure 3:**
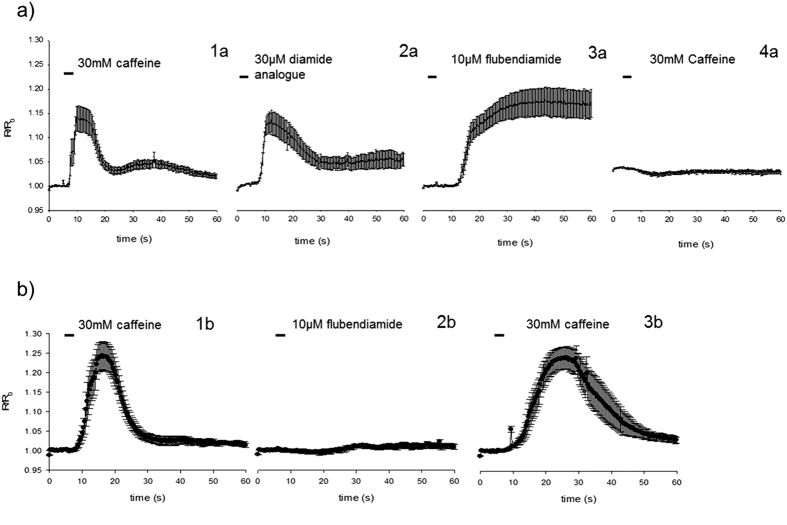
Fluorescence ratios measured over time in a single calcium release experiment using Fura-2 AM in Sf9 cells transiently expressing (**a**) WT construct and (**b**) the G4946E variant 72 h post transfection. Individual graphs for the WT construct show mean ± standard error responses of the same cells (n = 8) to 30 mM caffeine **1a,** 30 μM flubendiamide sulfoxide **2a,** 10 μM flubendiamide **3a** and 30 mM caffeine **4a**. Application of flubendiamide abolishes further caffeine responses. Individual panels for the G4946E construct show mean ± standard error responses of the same cells (n = 8) to 30 mM caffeine **1b**, 10 μM flubendiamide **2b** and 30 mM caffeine **3b.** Unlike in the WT construct the application of flubendiamide does not abolish further caffeine-mediated calcium release, suggesting a decreased binding affinity of flubendiamide to the G4946E construct.

**Figure 4:**
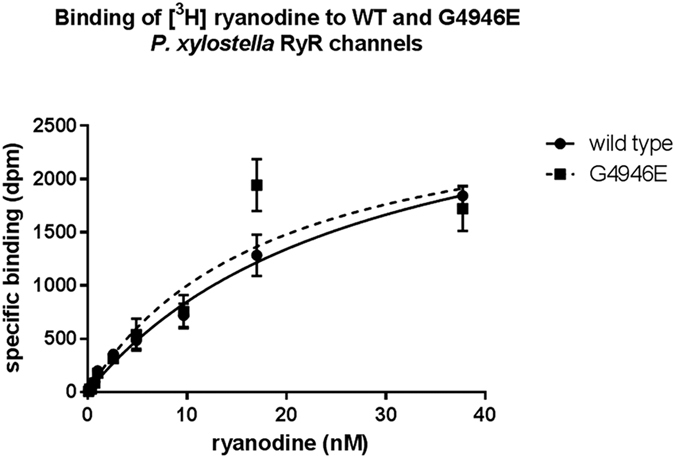
Tritium labelled ryanodine binding from Sf9 membrane preparations expressing *P. xylostella* WT and G4946E RyRs. The presence of the G4946E substitution does not have any marked effects on ryanodine binding capabilities.

**Figure 5:**
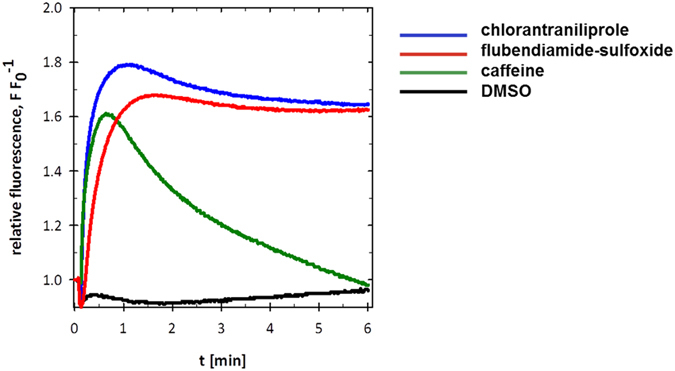
Calcium transients induced by diamides and caffeine in Sf9 cells expressing the *P. xylostella* ryanodine receptor. Sf9 cells expressing the wild-type ryanodine receptor were treated with 0.1% (v/v) DMSO (final conc.), 30 mM caffeine in 0.1% DMSO, 1 μM flubendiamide-sulfoxide, or 1 μM chlorantraniliprole in 0.1% DMSO (final conc.). Fluorescence of the calcium-Fluo8 complex was measured as a function of time.

**Figure 6:**
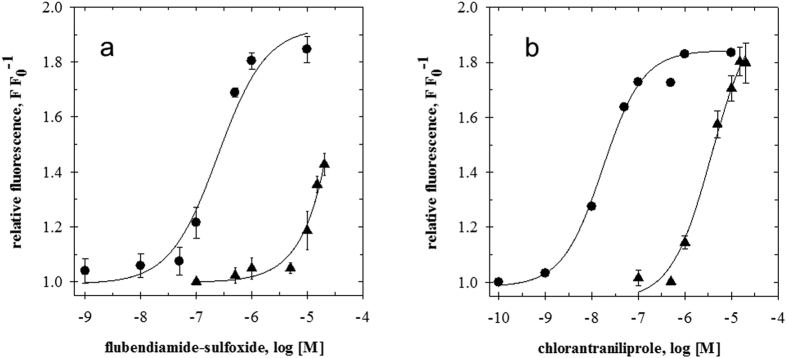
Calcium transients in Sf9 cells stably expressing either the *P. xylostella* WT (●) or the modified (G4946E) RyR (▲) as a function of the diamide concentration. Calcium-dye (Fluo8) fluorescence was measured after (**a**) flubendiamide-sulfoxide or (**b**) chlorantraniliprole application as a function of time. The integrated fluorescence signals were plotted against the logarithm of the effector concentration. Data fitting and EC_50_ calculations were performed with the SigmaPlot v13 software package.

For assembly of a full-length RyR ORF, four overlapping fragments (F1-F4) were amplified by PCR and cloned (Fig. 2a). Sequencing of individual clones for each of the four fragments revealed that the RyR obtained from the ROTH strain is polymorphic, with significant single nucleotide polymorphisms (SNP) between clones and alternative splice site variability (Fig. 2b). Due to the high number of SNPs, a *Pvu*I restriction site had to be engineered back into the F2 fragment selected for assembly into the full-length ORF. For the preparation of the RyR G4946E variant, the glutamic acid (E) was introduced into fragment F4 prior to final assembly of the full-length ORF. Initial assembly of both the full-length ROTH WT and G4946E RyR variant was achieved through simultaneous ligation of the 4 pre-digested fragments F1-F4 (in an equal weight ratio) into the vector pcDNA 3.1(-), between the *Not*I and *Kpn*I sites of the plasmids multiple cloning site (MCS). The final assembled ORF comprised 15357bp, coding for a 5118 amino acid protein The G4946E diamide resistance associated substitution^21^ is located at amino acid position G4900 in the expressed version of the *P. xylostella* channel. The full-length ORF was then moved across into the expression vector pIZ/V5-His, pre-modified by engineering an *Nru*I site at position 695 downstream of the MCS, within the V5 epitope. The full-length RyR ORF was excised from the pcDNA 3.1(-) plasmid using *Not*I and *Pme*I and ligated into the modified pIZ V5/His, between the *Not*I and *Nru*I sites.

Degenerate PCR amplifications were carried out using gradient PCR and REDtaq mastermix (Sigma) in 25 μl reactions consisting of 12.5 μl mastermix, 2 μl of each degenerate primer (20 mM), 1 μl cDNA and water. Cycling conditions were 95 °C for 2 min followed by: 35 cycles of 95 °C for 20 s, 45–50 °C for 45 s and 72 °C for 3 min with a final extension time of 72 °C for 5 min.

The initial long fragment PCR amplifications (25 μl) used Long Range PCR mix (Thermo) and consisted of 2.5 μl reaction buffer containing MgCl_2_, 1 μl cDNA, 1 μl dNTPs (10 mM), 0.5 μl of each primer (20 mM) and 2.5 U of enzyme mix. Cycling conditions were 94 °C for 2 min followed by: 35 cycles of 94 °C for 10 s, 50 °C for 20 s, 68 °C for 25 min, followed by a final extension of 68 °C for 15 min.

5′ and 3′ RACE used a First Choice® RLM RACE kit (Ambion®-Life Technologies) following the manufacturer’s protocol.

RyR fragments (F1-F4) for the final assembly were amplified using *pfu* proofreading polymerase (Promega) in 25 μl reactions consisting of 2.5 μl reaction buffer containing MgCl_2_, 1 μl cDNA, 1 μl dNTPs (10 mM), 0.5 μl of each primer (20 mM) and 1.5 U of enzyme. Cycling conditions were 95 °C for 2 min followed by 35 cycles of: 95 °C for 10 s, 50 °C for 20 s, 68 °C for 20 min and a final extension at 68 °C for 10 min.

The primer sequences for all of the cloning steps are shown in Tables 1, 2 and 3. All PCR amplified fragments were visualized on 1% (w/v) agarose gels and purified using a QiaQuick® gel extraction kit (Qiagen), following the manufacturer’s guide, prior to downstream applications.

All of the PCR-amplified fragments were ligated into a pJET 1.2 blunt end vector (part of CloneJET cloning kit (Thermo)) following the kit’s protocol. Fastdigest® restriction enzymes (Thermo) were used for all restriction digests, following the manufacturer’s instructions. The full-length ligations into pcDNA 3.1(-) and pIZ/V5-His were done in 30 μl reactions, using 3 μl of 10 × ligation buffer and 10U of T4 DNA ligase (Thermo) for 2 h at room temperature. All plasmids were transformed into XL-10 gold *E. coli* (Agilent), recovered and grown at 30 °C to minimize potential rearrangements within the plasmid. Transfections grade plasmid preps were obtained using a Plasmid maxi prep *plus* kit (Qiagen) following the manufacturer instructions. All mutagenesis reactions were done using a Quikchange II mutagenesis kit (Agilent) following the kit’s manual (mutagenesis primers are shown in Table 4).

### Sf9 Transfections and membrane preparation

Sf9 cells (Life technologies) were maintained in Sf-900 II SFM (Gibco-life technologies) serum free media without antibiotic in 30 ml suspension cultures (shaking at 115 rpm) at a constant temperature of 27 °C. Cells used for transfection were seeded into 60 mm cell culture dishes (Corning^®^), at 2 × 10^6^ cells per dish in 3 ml of Sf-900 II SFM, and left to attach for 1 h. Cells were transfected using 3 μg of plasmid DNA, 15 μl Cellfectin II reagent (Life technologies) and 5 μl PLUS™ enhancer reagent (Life technologies) per dish, following the manufacturer’s protocol.

For membrane preparation, transfected cells were harvested 48 h post transfection using phosphate buffered saline (PBS), the cells counted and then re-suspended in a hypo-osmotic buffer (20 mM Tris, 1 mM EDTA pH 7.4) at a concentration of 2 × 10^6^ cells per ml. The cells were then homogenised and centrifuged at 1,500 g for 15 min at 4 °C to remove cell debris. The supernatant was removed, centrifuged at 100,000 g for 1 h at 4 °C and the membrane pellet re-suspended in a 0.4 M sucrose, 20 mM HEPES solution (20 μl per 2 × 10^6^ cells). Protein concentration was determined using Bradford reagent (Bio-Rad) following the manufacturer’s instructions, against Bovine Serum Albumin (BSA) standards.

### Calcium release assays

Fura 2-AM dye (Life technologies, CA, USA) was used for monitoring calcium release in Sf9 cells transfected with the *P. xylostella* RyR. Approximately 2.5 × 10^5^ of un-transfected SF9 cells were allowed to attach to Poly-L-lysine coated coverslips (Sigma, MA, USA) for 1 h and then transfected using Cellfectin™ II. 24 h, 48 h, 72 h and 96 h post transfection the cells were loaded with 1 mM of Fura 2-AM calcium sensitive dye. Cells were left to incubate at 27 °C for 45–60 minutes, followed by 3 washes with 500 μl of fresh un-supplemented Sf-900 II medium. Prior to imaging, coverslips with Fura 2 loaded cells were placed in standard Ringer’s solution. All imaging was done using an Axio Vert.A1 microscope with a LD Plan-Neo Fluar ×20/0.4 lens (Zeiss, Oberkochen, Germany), measuring the ratio of excitation at 340/380 nm (calcium free/calcium bound indicator) every 180 ms and capturing emission at 510 nm for at least 60 seconds. Cells on coverslip were placed into a perfusion chamber of approximately 0.5 ml volume mounted on the microscope stage which was connected to a peristaltic pump, allowing for a constant fluid exchange (flow rate 3 ml/min). Test solutions were applied using 3–5 seconds bursts via a glass u-tube. Experiments were recorded using VisiView^®^ software (Visitron Systems, Puchheim, Germany) and the numerical data analysed using Microsoft Excel 2010 and SigmaPlot v.12 (Systat Software). The data were normalized using the equation: R/R_0_, where R is a fluorescence ratio value recorded for individual time points and R_0_ is an average fluorescence ratio calculated over the first 5 seconds prior to addition of the agonist. The normalized amplitude responses of individual cells were calculated by estimating the highest value subtracted from the basal level (R_max_–R_0_ = ∆R_max_) and normalizing with the equation: ∆R_max_/R_0_. Final amplitude data was presented as a mean value of all cells in the individual experiment and the standard deviation of the mean.

### [^3^H] Ryanodine binding

Binding reactions were set up in a 96 well flat bottom microtiter plate in a total volume of 250 μl per well. Reactions consisted of either 25 μg of membrane prep, [^3^H] ryanodine (concentrations 0.01–40 nM) and 2 μl of DMSO in a binding buffer containing 0.01% Pluronic (1.5M KCl, 10 mM ATP, 1.38 mM CaCl_2_, 10 mM HEPES pH 7.4) or 25 μg of membrane prep, [^3^H] ryanodine (0.01–40 nM), 2 μl of ryanodine in DMSO (final concentration 10 μM) in 0.01% Pluronic. The reactions were incubated at room temperature for 2 h. The 96 samples were then loaded onto a 96 well filter plate pre-treated with 50 μl of 0.1% polyethylenimine, using a 96 well plate harvester (Brandel, MD, USA). Each filter with bound membranes was then washed 3 times with 250 μl of wash buffer (150 mM KCl, 10 mM HEPES pH 7.4 and left overnight at room temperature to dry. Each well on the plate was then filled with 50 μl MicroScint™-O (PerkinElmer, MA, USA) and the filter plate loaded into a TopCount NXT™ Micro-plate Scintillation counter (PerkinElmer, MA, USA). Specific binding and binding kinetics values (equilibrium dissociation constant K_d_ and Binding capacity/receptor density B_max_) were calculated using GraphPad Prism v5.5 (GraphPad, CA, USA).

### Selection of stable cell lines

Cells were transfected as described above and subjected to selection with 300 μg ml^−1^ zeocin by the dilution method, essentially as described in the manufacturer´s manual (Invitrogen). After a few successive rounds of clonal selection, zeocin-resistant cell clones were tested for functional RyR expression by calcium fluorescence measurements.

### Calcium fluorescence measurements

Calcium-dye fluorescence was measured with Fluo8-loaded cells on the FLIPR Tetra instrument (Molecular Devices) in the 384 microtiter plate format. 1.5 × 10^4^ cells per well were treated with variable concentrations of effector compounds in a total volume of 80 μl Tyrode´s buffer containing 0.1% DMSO (final concentration) essentially following the established protocols provided with the Fluo8 No-Wash calcium assay kit (Molecular Devices). Excitation and emission wavelengths were set at 490 nm and 525 nm, respectively.

## Additional Information

**Accession codes:** AFW97408.

**How to cite this article**: Troczka, B. J. *et al.* Stable expression and functional characterisation of the diamondback moth ryanodine receptor G4946E variant conferring resistance to diamide insecticides. *Sci. Rep.* **5**, 14680; doi: 10.1038/srep14680 (2015).

## Figures and Tables

**Table 1:** Degenerate primers.

**Primer Name**	**Sequence 5′-3′**	**position**
Px.NAdf	TTYYTNCGDACSGAAGAYATGG	close to 5′ end
Px.N1dr	GGTWTNGTNARRCAYTCRTCDCC	
Px.CAdf	GGARTTYGACGGNCTGTWCATYGC	close to 3′ end
Px.C1dr	TCWCCNACNGGGAARAAGTCCC	
Px.MBdf	GACTTCCTGAGVTTYTGYGTTTGGG	middle of ORF
Px.M2dr	GCRTAGTTCTCRGCYTCGTTGTAGA	

**Table 2:** RACE primers.

**Primer name**	**Sequence 5′-3′**	**position**
Px.3′RACE inner-1F	GAGTCCAATTGCTTCATCTGTGGC	15103–15126
Px.3′RACE outer-2F	CATCGGAGACGAACTGGAGCC	14928–14948
Px.5′RACE inner-1R	CGCTCAGTAGCGACAGACACGAGG	498–521
Px.5′RACE outer-2R	GTACCGCTCAGTAGCGACAGACAC	502–525
Px.5′RACE F	CATGGTGTGCCTGTCCTGCACG	63–84

**Table 3:** Primers for amplification of RyR fragments F1–F4.

**Primer name**	**Sequence 5′-3′**	**Position**
Px.F1-F	CAAAATGGCGGAAGCGGAAGGGGGTGCG	−4–24
Px.F1-R	TCCTCTTGACCGTCATCATAGTCGCGG	4071–4097
Px.F2-F	GCGCACATAGACCAGATCATGAGGAGC	4012–4038
Px.F2-R	CGTCAAGGAATTGGATGAAGAGCCTAAAGC	6435–6464
Px.F3-F	GGCACTGCAAGAGTCTGAAGTTGAGG	6348–6373
Px.F3-R	CCAATACCAAGGCTACAAGCTACACAGG	9105–9132
Px.F4-F	GTGCCCTACGACCTGCTAACGGAC	9131–9154
Px.F4-R	GACTTAGGGCCACTGGTACCAAACTC	15357–15382

**Table 4:** Mutagenesis primers.

**primer**	**Sequence 5′-3′**	**description**
Px.G4946E-mut-F	GGACGTGGCTGTTGAGTTCAAGACGTTGAGG	G4946E primers
Px.G4946E-mut-R	CCTCAACGTCTTGAACTCAACAGCCACGTCC	
pIZ/His-NruI SDM-F	CCCTCTCCTCGGTCGCGATTCTACGCGTACC	pIZ NruI site primers
pIZ/His-NruI SDM-R	GGTACGCGTAGAATCGCGACCGAGGAGAGGG	
